# HPV self-sampling as an alternative strategy in non-attenders for cervical screening – a randomised controlled trial

**DOI:** 10.1038/bjc.2011.48

**Published:** 2011-02-22

**Authors:** A Szarewski, L Cadman, D Mesher, J Austin, L Ashdown-Barr, R Edwards, D Lyons, J Walker, J Christison, A Frater, J Waller

**Affiliations:** 1 Cancer Research UK Centre for Epidemiology, Mathematics and Statistics, Wolfson Institute of Preventive Medicine, Barts and The London School of Medicine and Dentistry, Queen Mary University of London, Charterhouse Square, London EC1M 6BQ, UK; 2 St Mary's Hospital, Praed Street, Paddington, London W2, UK; 3 NHS Westminster, 15 Marylebone Road, London NW1 5JD, UK; 4 South Central Strategic Health Authority, Rivergate House, London Road, Newbury, Berkshire RG14 2PZ, UK; 5 Department of Epidemiology and Public Health, Cancer Research UK Health Behaviour Research Centre, UCL, Gower Street, London WC1E 6BT, UK

**Keywords:** HPV, screening, self-sampling

## Abstract

**Background:**

A randomised trial to ascertain whether women who do not attend for cervical screening are more likely to respond to the opportunity to collect a self-sample for human papillomavirus (HPV) testing, or to a further invitation to attend for cervical screening.

**Methods:**

The study was carried out in a Primary Care Trust (PCT) in London between June 2009 and December 2009. In total, 3000 women were randomly selected from persistent non-responders (i.e., who had not responded to at least two invitations to attend for screening). The women were randomised on a 1 : 1 basis to either receive an HPV self-sampling kit or a further invitation to attend for cervical cytology. The main outcome measures were (1) percentage of women attending for cervical cytology compared with those returning a self-sample HPV test or attending for cytology subsequent to receiving the kit and (2) percentage of those testing positive for HPV who attended further investigation.

**Results:**

The total response in the self-sampling group for screening was 10.2%. Of the 1500 women in the control group sent a further invitation for cervical screening, 4.5% attended for cytology screening. This difference is highly statistically significant (*P*<0.0001). Of the 8 women who tested positive for HPV, 7 attended for a cervical smear and had a concurrent colposcopy. Three of these (43%) had high-grade disease (defined as CIN 2+), with one found to have an invasive cancer (stage 1b) and one CIN 3.

**Conclusions:**

The value of this intervention relies on the detection of high-grade CIN and early stage cancer with a good prognosis. The relatively high yield of abnormalities found is consistent with that expected among a hard to reach and relatively high-risk group of women. Our study suggests that self-sampling could increase participation among non-responders in England, but further work is needed to ascertain whether the low response rate seen here is likely to be representative of the rest of the country. Other studies are needed to investigate the response to self-sampling in different demographic and geographic settings.

Attendance for cervical screening is falling in England, particularly among women aged 25–29 years (NHS CSP, 2006; Willoughby *et al*, 2006; Lancuck *et al*, 2008). This is of concern as screening has been demonstrated to reduce the incidence of cervical cancer yet coverage of the screening programme has now fallen below its stated target of 80%. Uptake is considered to be the most important factor in determining the success of a screening programme (Weller and Campbell, 2009). Non-attenders are at higher risk of cervical cancer; therefore, encouraging such women to take part would save lives and the costs of invasive cancer treatment. Deprivation and being from an ethnic minority group are both associated with poor uptake of screening (Webb *et al*, 2004; Downs *et al*, 2008; Moser *et al*, 2009). An NHSCSP survey (NHS CSP, 2006) and a recent population-based survey (Waller *et al*, 2009) have both identified practical issues (such as taking time off for appointments) as being important in influencing women's attendance for screening. These may even be more important than the emotional issues previously identified as barriers, such as embarrassment, wish for a female doctor and discomfort associated with the test (NHS CSP, 2006; Baron *et al*, 2008; Waller *et al*, 2009; Wilson, 2009).

One way of increasing coverage and overcoming some of the practical and emotional barriers to screening attendance might be to use self-sampling for high-risk human papillomavirus (HPV) types as a primary screening test for non-attenders. Human papillomavirus self-sampling has similar sensitivity and specificity to samples taken by clinicians (Petignat *et al*, 2007; Szarewski *et al*, 2007), and we have shown that it is broadly acceptable to women (Forrest *et al*, 2004; Waller *et al*, 2006; Szarewski *et al*, 2009). There have been only four published studies looking specifically at self-sampling in screening non-attenders carried out in the Netherlands (Bais *et al*, 2007; Gok *et al*, 2010), Sweden (Sanner *et al*, 2009) and Italy (Giorgi Rossi *et al*, 2011). In the Scandinavian studies, 34%, 27% and 39%, respectively, of non-attenders sent back a self-sample kit which had been posted to them, compared with 17% in the Dutch study who attended for screening following a further invitation (in Sweden there was no control group). However, in the study in Italy, the response was considerably lower, ranging between 8.7 and 19.6%, depending on the exact intervention.

If self-sampling does prove acceptable to non-attenders, it would be possible to make it widely available. There are already laboratories all around the country which are able to carry out HPV testing, and it is likely that the numbers and capability will increase in the near future as HPV testing for the triage of borderline/mildly abnormal cytology is rolled out from the sentinel sites. As the samples can be posted to the laboratory, the service does not have to be available at every hospital, but can be concentrated in those which have the expertise and capacity.

The cervical screening unit in Westminster Primary Care Trust (PCT) was set up in 1990. It is responsible for the call, recall and management of results for the 62 000 eligible women in the area. Westminster PCT is ethnically and culturally diverse, with 27% of the population classified as non-white (ONS, 2010). Cervical screening coverage in Westminster in 2008–2009 was 68.1% overall, 59.7% in the age group 25–49 years and 70.4% in the age group 50–64 (The Health and Social Care Information Centre, 2009).

The aim of this study was to ascertain whether women who do not attend for cervical screening are more likely to respond to the opportunity to collect a self-sample for HPV testing, or to a further invitation to attend for cervical screening.

In addition, it is important to ascertain whether such women will attend for further investigation, if they have a positive screening test (HPV test or cervical cytology). It would be of little value if the women were persuaded to undergo screening, but then refused further investigation and treatment.

## Subjects and methods

This study was carried out in 2009 using the Exeter National Health Applications and Infrastructure Services (NHAISs) System in Westminster PCT. In all, 3000 women were randomly selected from those identified as persistent non-responders (i.e., had not responded to at least two invitations to attend for screening, normally the number sent in the screening programme). These women were then randomised on a 1 : 1 basis to either receive an HPV self-sampling kit or a further invitation to attend for cervical cytology.

Women randomised to self-sampling were sent a mailing in June 2009 from the PCT containing an initial contact letter, a patient information leaflet about the study and consent form in duplicate (one copy to be returned), an information sheet on how to perform the test, an HPV information leaflet and the testing kit. The control group were sent a standard PCT invitation letter for cervical cytology screening. Both groups received a questionnaire survey that collected demographic and psycho-social information, as well as reasons why they had not attended for screening. They were asked to complete and return it whether or not they took any further action. Study information was available (both as hard copy and on the Internet) in Cantonese, Arabic, Farsi, Bengali and Portuguese, identified as the most prevalent non-English languages in the area. Women who responded within 6 months of the start date were included in the analysis.

Women were provided with written and pictorial instructions detailing how to carry out the self-sampling test. They were also provided with pre-paid, addressed packaging for the return of the samples. The self-sampling kit used was the Qiagen sampler (QIAGEN Ltd, West Sussex, UK), which utilises a cotton swab. Human papillomavirus testing was carried out using the Qiagen hybrid capture II (HC-II) test. Results were recorded in relative light units compared with a 1.0 pg ml^−1^ standard. A positive HPV result was defined as a value greater or equal to the standard threshold of 1.0 pg ml^−1^.

Women who had a positive HPV test were invited to attend for cervical cytology at a dedicated clinic, held in the colposcopy unit at St Mary's Hospital, London. When they attended for cervical cytology, they were offered the option of a colposcopy immediately following (i.e., at the same visit as) their cytology screen, having been informed of this choice in advance. The rationale for this was to save them from having to return for a further gynaecological examination at a later date should they have an abnormal result. In addition, those who had a positive HPV test but negative cytology would have the immediate reassurance of a colposcopy and were also given the opportunity to discuss the implications in advance.

Women in the control group who had a cervical cytology result showing mild dyskaryosis or worse were invited for colposcopy at St Mary's Hospital, in accordance with routine practice. The NHAIS Open Exeter database was used to check attendance by all women contacted (in both groups) for cervical screening and results as appropriate.

The study was funded by a Research for Patient Benefit (RfPB) Programme grant: PB-PG-0407-13358 and was approved by the St Mary's Research Ethics Committee.

### Statistical analysis

A conservative assumption was made, based on the only comparable published study at the time of preparation (Bais *et al*, 2007), of response rates of 15% in the self-sampling group and 7% in the control group, allowing for 15 and 7% positivity by HPV and cytology respectively, and allowing for 20% of women on GP lists to be ‘ghosts’ (i.e., letters sent to an incorrect address). Thus it was calculated that 1500 women would be needed in each group to achieve 83% power to detect a significant difference at the 5% significance level between the two groups with regard to the total proportion responding.

Women in the arm sent a further invitation for screening were considered to be responders if they attended for cervical screening between the date of being sent the invitation and up to 6 months after this date. Women in the self-sampling arm were considered to be responders if a self-sampling test had been returned within 6 months. We also identified women who had not returned a self-sampling kit but attended for cervical screening between the date of being sent the self-sampling kit and up to 6 months after this date.

We compared the percentage of those attending cervical screening in the invitation arm and the percentage of those either returning a self-sample or attending for cervical screening in the self-sampling arm using a 2 × 2 tabulation and Pearson's *χ*^2^-test with one degree of freedom.

Initially, we compared deprivation between responders and non-responders in the two groups. Deprivation is calculated based on UK postcodes. Postcodes are categorised into Q1 (most deprived quintile in the UK) to Q5 (least deprived quintile in the UK) (data downloaded from GeoConvert: http://geoconvert.mimas.ac.uk/). Age was compared between the two groups using a Wilcoxon rank sum test.

Cytology and colposcopy results were taken from the first cytology resullt recorded after date letter sent (if the intial result was unsatisfactory, we present the repeat cytology result where available). Histology is presented as the worst result of the punch biopsy and LLETZ specimen where taken.

## Results

**Figure 1:**
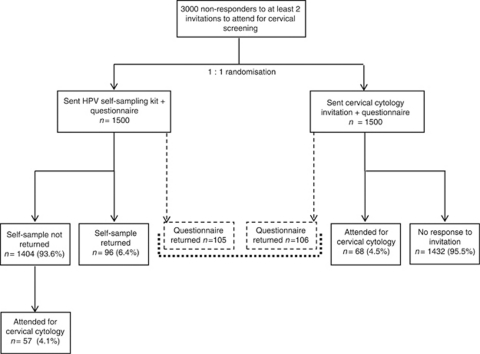
Overview of study design.

Figure 1 (flow chart) shows the study design and number of women who responded. Of 1500 women who were sent a self-sampling kit, 96 (6.4%) returned a self-sample and 105 (7%) returned a questionnaire (93 returned both a self-sample and questionnaire). A further 57 women (3.8%) sent a self-sampling kit who did not return it attended for a routine cervical screen. Therefore, the total response in the self-sampling group for screening was 10.2%. Of the 1500 women in the control group sent a further invitation for cervical screening, 68 (4.5%) attended for cytology screening between June 2009 and December 2009. This difference is highly statistically significant (*P*<0.0001). In all, 106 of the women in the cytology screening group (7%) returned a questionnaire.

**Figure 2:**
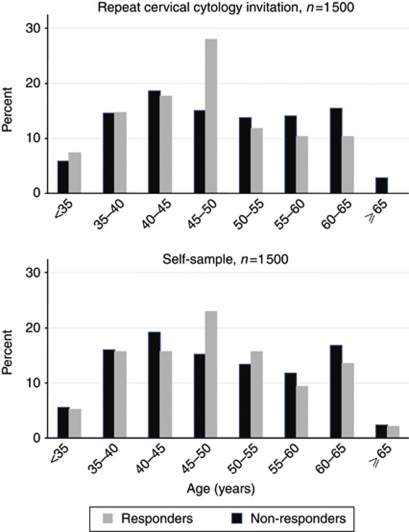
Age distribution of 3000 women in the study.

The median age overall was 48 years, with a range of 29–65 years (IQR 41–57 years). There was little difference in age distribution between responders and non-responders in the two groups (Figure 2): in the self-sampling group, the median age for responders was 47.5 years (IQR 40.5–54.5 years), while for the non-responders the median age was 47 years (IQR 40–57 years), *P*-value=0.68. In the further invitation for cervical cytology group the median age for responders was 46.5 years (IQR 40–52 years), while for the non-responders the median age was 48 years (IQR 41–57 years), *P*-value=0.19. The overall proportion of women under the age of 35 years was 5.7% and was similar in all groups. Table 1 compares the social deprivation scores in the two groups; it can be seen that there is little difference between them.

For those 96 women who returned a self-sample, 95 (99.0%) were adequate (one sample tube arrived containing no liquid). In all, 8 out of 95 (8.3%) had a positive HC-II result. One woman did not respond to the subsequent invitation to attend for cervical cytology. She confirmed her attendance at a private clinic for cervical cytology subsequent to her HPV test, and that the result was negative. The remaining seven women attended for cervical cytology and all of them took up the offer of an immediate colposcopy. Table 2 shows the outcome of cytology and histology. Where women had both a punch biopsy and a loop excision (LLETZ), the worst histology is given. Taking the worst histology result, 3 (43%) had high-grade disease (defined as CIN 2+), with one found to have an invasive cancer (stage 1b) and one CIN 3.

Of the 1404 women sent a self-sample kit who did not return a self-sample, 57 attended for cervical cytology within the following 6 months (i.e., 57 out of 1404 or 4.1%). In all, 50 (87.7%) had a negative cytology result, 2 (3.5%) had borderline/mild dyskaryosis and in 5 cases (8.8%) the sample was inadequate for diagnosis.

In all, 68 of the 1500 (4.5%) women sent a further invitation for cytology screening (the control group), attended. Table 3 shows their results. One woman was found to have severe dyskaryosis; she had CIN2/3 on punch biopsy and CIN3 on the loop specimen.

### Reasons for non-attendance

In total, 211 questionnaires were received (i.e., a 7% response rate). In total, 105 were from women randomised to receive a self-sample kit and 106 were from those randomised to receive a further invitation for cytology screening. In total, 180 of the women gave at least one reason for previous non-attendance for screening. A number of themes were apparent (Table 4), which related to the unpleasant/embarrassing nature of the examination, practical issues such as lack of time, problems with transport, making appointments and perceptions of being at low risk for disease.

Thirty women in the self-sampling group and 39 in the control group had actually attended for cytology screening in the 3 months before the study, but their results had not yet been entered onto the system when the study letters were generated.

## Discussion

The response rate in Westminster PCT in this study was considerably lower than in the three studies of non-responders in the Netherlands and Sweden (Bais *et al*, 2007; Sanner *et al*, 2009; Gok *et al*, 2010). Those studies showed response rates for HPV self-sampling of 34, 39 and 27%, respectively, whereas the response rate in this study to self-sampling was 6.4%, with a further 3.8% of those sent self-sampling kits attending for cytology screening. This brings the response rate in the self-sampling group to 10.2% compared with 4.5% in the cytology screening group. Only 7% of women returned the questionnaire in both the study and control groups. The response rate in the Italian study (Giorgi Rossi *et al*, 2011), while lower than in the other studies, was still higher than in Westminster PCT. However, it should be noted that the population in the Italian study was somewhat different, in that the women (aged 35–65 years) were eligible when they had missed only one invitation, and were <5 months late for screening attendance.

This study was designed to adhere as closely as possible to current practice within the NHS cervical screening programme and to have a similar intervention level in both the study and control arms. In contrast, the non-responders in the studies in Sweden and the Netherlands were contacted more than once to encourage them to participate. In addition, despite non-attendance being identified as a particular problem in the UK in women under 35 years, in this study under 35s accounted for <10% of the population. This is because we chose to target women who had not responded to at least two invitations; if screening starts at 25 years and is rolled out over 3 years, women will be well over 30 years by the time they fall into this category. The timing of this study coincided with a high level of publicity regarding the death from cervical cancer of the celebrity Jade Goody. This resulted in increased levels of attendance for cervical screening throughout the country (Bowring and Walker, 2010). This may have encouraged some non-attenders to go for the established cytology test, rather than to perform a self-sample. Indeed, 3.8% of those in the self-sampling group (57 out of 1500) attended for cytology rather than sending back a self-sample. However, it is unlikely that this was a major factor, as the uptake of cervical screening in the control arm of the study was only 4.5%. Westminster PCT has a high level of population mobility, with an annual population turn over of 390 per 1000 registered GP patients in 2007–2008 (Guy, 2009). As a result, it is not possible to be certain that invitations actually reached the women invited. In the studies in Sweden and the Netherlands it is likely that the population was better defined due to the presence in both countries of identity numbers. In addition, those studies excluded women who were not eligible for routine screening, for example, those who had undergone hysterectomy. In the UK, this information is dependent on the patient being removed by GPs from the Patient Notification Lists, and their accuracy is variable.

Unfortunately the 7% response rate to the return of the questionnaires is too low to be considered representative. Although the study information was available in other languages (and this was stated in these languages in the information sent), we did not receive any requests for translations. In all, 90% of women who returned a questionnaire stated that they had lived in the UK for >10 years and that their preferred language was English. The way information is presented can be very important in influencing women's decisions regarding screening (Giordano *et al*, 2008; Weller and Campbell, 2009). However, it is interesting to note, in relation to those women who chose to respond, that the prevalent themes were similar to those identified in a recent population-based interview study of 580 women in England (Waller *et al*, 2009). In that study, the most endorsed barriers to attendance for screening were embarrassment with regard to the examination, fear of what the test might find, fear of pain, lack of time or not getting around to attending, perceived difficulties in arranging a convenient appointment and having had a bad screening experience in the past. Self-sampling for HPV could be a way of removing some of these barriers (e.g., the embarrassment, the fear of pain and the time required to go to a clinic) and may indeed be a solution for some, but not all women (Forrest *et al*, 2004; Szarewski *et al*, 2009). Cultural sensitivities need to be taken into account and, given the low response rate in this study, more studies in the UK are needed before any decisions are taken regarding implementation of such a strategy.

The HPV positivity rate in this study, at 8.2%, was similar to that found in a screening study in a comparable age group in the UK (Cuzick *et al*, 2003), in which the HC-II test was used. In England and Wales, approximately one woman in 10 000 screened is found to have invasive cancer (NHS CSP, 2009).

In this study, 8 out of 96 women screened by self-sampling tested positive for high-risk HPV. Seven of these women attended for colposcopy, high-grade disease was detected in three (43%) with one found to have CIN 3 and one an invasive cancer (stage 1b).

In the study by Giorgi Rossi *et al* (2011), it was calculated that 13 self-sampling kits had to be sent out for every one that was returned from women who had not attended for screening within 3 years (the subgroup closest to this study). In Westminster PCT, the corresponding number is 16. We have estimated that the administrative, laboratory and colposcopy costs of our study (i.e., not specifically related to the research aspects) could be as much as £13k for the self-sampling group, but only if using a commercial rate for the HPV tests of £42 per test. It is anticipated that the cost of HPV testing will decrease once it is adopted into the NHS for triage of borderline cytology and follow-up of women who have been treated for CIN. Indeed, this is already ongoing in the sentinel sites (NHS CSP, 2011), and is due to be rolled out nationwide during 2011. Nevertheless, this study suggests that savings of at least this amount can be expected by increasing the uptake of screening among high-risk women. Even on the conservative assumption of between two and three cancers prevented among the six additional abnormalities detected, savings exceeding £15k are anticipated applying the costs of the care pathway for screen detected cancers set out in the ARTISTIC study (Kitchener *et al*, 2009). The value of this intervention relies on the detection of high-grade CIN and early stage cancer with a good prognosis. The relatively high yield of abnormalities found is consistent with that expected among a hard to reach and relatively high-risk group of women. The albeit limited success of the approach would certainly seem to merit further investigation.

Our study suggests that self-sampling could increase participation among non-responders in England, but further work is needed to ascertain whether the low response rate seen here is likely to be representative of the rest of the country. Other studies should now be carried out to investigate the response to self-sampling and its cost effectiveness in different demographic and geographic settings.

## Figures and Tables

**Table 1:** Comparison of social deprivation scores in the two groups (ascertained by postcode)

	**Self-sampling group**			**Cervical cytology group**		
	**Responders**	**Non-responders**	**Total**	**Responders**	**Non-responders**	**Total**
Q1 (most deprived)	23 (5.1%)	430 (94.9%)	453	16 (4.0%)	386 (96.0%)	402
Q2	26 (6.6%)	367 (93.4%)	393	17 (4.0%)	403 (96.0%)	420
Q3	23 (6.4%)	336 (93.6%)	359	17 (4.5%)	357 (95.5%)	374
Q4	24 (8.3%)	264 (91.7%)	288	18 (6.1%)	278 (93.9%)	296
Q5 (most affluent)	0 (0.0%)	7 (100.0%)	7	0 (0.0%)	8 (100.0%)	8
Total	96	1404	1500	68	1432	1500

Responders are defined as women returning a self-sample or attending for cytology screening (not whether they returned a questionnaire).

Crown Copyright 2006.

Source: National Statistics/Ordnance Survey; extracts are Crown Copyright and may only be reproduced by permission.

**Table 2:** Histology results in the women who tested HPV positive

	**Worst histology**						
**Cytology**	**No biopsy taken**	**HPV only**	**CIN 1**	**CIN 2**	**CIN 3**	**Invasive cancer stage 1B**	**Total**
Negative	2	1	—	—	—	—	3
Borderline dyskaryosis	—	—	—	1	—	—	1
Moderate dyskaryosis	*—*	*—*	1	—	—	—	1
Severe dyskaryosis	*—*	*—*	*—*	—	1	1	2
Total	2	1	1	1	1	1	7

Abbreviations: CIN=cervical intraepithelial neoplasia; HPV=human papillomavirus.

**Table 3:** Cytology results of the 68 women who responded to the further invitation to attend for cytology screening

**Cytology result**	**Number (%)**
Inadequate	3 (4.4%)
Negative	62 (91.2%)
Borderline dyskaryosis	2 (2.9%)
Severe dyskaryosis	1 (1.5%)
Total	68

**Table 4:** Reason for non-attendance of cervical screening: taken from questionnaire

**Reason**	***n***
*Emotional or attitudinal reasons*	
Uncomfortable/painful/unpleasant/sexual abuse/dislike doctors or nurses	62
Embarrassed	48
No recent sexual activity/no problems/too old or young (incorrect perception of risk)	23
Putting off/not important/forgetting	22
Frightened to be told had cancer	18
Other	6
	
*Practical reasons*	
Lack of time/too busy/no child care/access to clinic/times/transport/disabled	49
Never had sex	17
Hysterectomy/operation – unnecessary	9
Attend elsewhere (abroad/privately)	8
Other	3
	
*Unknown*	
Other	9
	
Total	274
